# Coexistence and management of COVID-19 pandemic with other epidemics in West Africa: lessons learnt and policy implications

**DOI:** 10.11604/pamj.2021.38.341.27901

**Published:** 2021-04-08

**Authors:** Virgil Kuassi Lokossou, Denis Bunyoga, Issiaka Sombie, Stanley Okolo

**Affiliations:** 1 Economic Community of West African States (ECOWAS), Regional Center for Surveillance and Disease Control, Abuja, Nigeria; 2 West African Health Organization, Bobo-Dioulasso, Burkina Faso,; 3 Africa Center for Disease Control and Prevention, Addis Ababa, Ethiopia

**Keywords:** Coexistence, epidemics, COVID-19, pandemic, West Africa, infectious disease

## Abstract

Since the beginning of the COVID-19 pandemic in West Africa, the region has faced a coexistence of epidemics raising questions about the management of the coexistence between COVID-19 and other epidemic prone diseases. We undertook a cross-sectional study covering the period from February to August 2020 in which an extensive desk review was completed and questionnaire was submitted to National Public Health Institutes. In addition, we conducted online interviews with 10 West African countries to discuss in-depth the strategies and challenges in managing the coexistence of epidemics. Eight epidemics coexisted with COVID-19 in West Africa. These epidemics were yellow fever and measles in five countries; meningitis in 4 countries; vaccine derived poliomyelitis and dengue fever in two countries; Lassa fever, Crimean Congo Hemorrhagic fever and hepatitis E virus in one country. COVID-19 pandemic has brought both positive and negative effects to the management of other epidemics. The management of coexistence was similar in most ECOWAS countries with different incident management systems set up to manage separate outbreaks. The experience in managing the coexistence of epidemics led ECOWAS Regional Center for Surveillance and Disease Control to recommend to member states that they should reinforce regular disease surveillance for seasonal outbreaks and country specific epidemiological diseases profile while not forgetting other emerging and remerging infectious diseases.

## Commentary

The West African region is recognized as a zone with the recurrence of infectious diseases outbreaks such as meningitis, Lassa fever, cholera, yellow fever, Ebola etc. [1-7]. COVID-19 was first reported in West Africa in the first quarter of 2020 and reached all the 15 countries by April 2020 [8-10]. With regards to COVID-19 and other epidemic prone diseases management in West Africa, several questions remain and are the reflections of this commentary: i) what is the coexistence between the recurrent epidemic prone diseases and the COVID-19 pandemic? ii) how are West African countries managing COVID-19 pandemic and the other epidemics at the same time? and iii) what is the influence of the management of COVID-19 pandemic on other epidemics management in West Africa? This manuscript intends to present reflections to these questions.

Our responses have been elaborated on the basis of a cross sectional study in which questionnaires were answered and online meetings held with National Public Health Institutes of 10 ECOWAS countries namely: Burkina Faso, Cabo Verde, The Gambia, Ghana, Côte d'Ivoire, Niger, Nigeria, Senegal, Sierra Leone and Togo. These meetings, jointly organized by the ECOWAS Regional Center for Surveillance and Disease Control (RCSDC), West African Health Organization (WAHO) and Africa Center for Disease Control and Prevention (Africa CDC), aimed at promoting experience sharing, good practices and lessons learnt in managing the coexistence of COVID-19 pandemic and other epidemic prone diseases. They were attended by national public health institutes leaders, international health regulations national focal points, laboratory officers, public health experts, non-governmental organizations and other epidemics first line responders in West Africa. Prior to the data collection, an extensive desk review including epidemiological bulletins, events and indicator based surveillance reports from ECOWAS countries and regional organizations such as the Regional Center For Surveillance And Disease Control (RCSDC), West African Health Organization (WAHO), Africa CDC, the World Health Organization - Regional Office for Africa and other regional bodies was done.

**Coexistence of COVID-19 with other epidemics:** between February and August 2020, 8 epidemics coexisted with COVID-19 in ten ECOWAS countries. These epidemics were yellow fever and measles in five countries; meningitis in 4 countries; vaccine derived poliomyelitis and dengue fever in two countries; Lassa fever, Crimean Congo Hemorrhagic fever and hepatitis E virus in one country. Some countries experienced more than one disease epidemics with five in Burkina, Nigeria and Niger, four in Ghana and Senegal and two in Sierra Leone. We also found that the number of epidemic disease cases reported by the countries, were fewer in 2020 compared to previous years during the same period. The decreasing number of confirmed cases of other epidemics reported during the COVID-19 pandemic might be due to overwhelmed surveillance system and the limited capacity for detection in West Africa. This observation was confirmed by the laboratory network records reporting the decrease of number of samples collected.

**Management of COVID-19 pandemic and other epidemics:** the management of coexistence was similar in most ECOWAS countries with different incident management systems set up to manage separate outbreaks. Ivory Coast was the unique country reporting one incident management system managing all ongoing epidemics. Having one Incident Management and Systems was really helpful to implement synergistic public health interventions during the epidemics responses and to make efficient use of available resources during the response. COVID-19 tools for surveillance, laboratory and risk communication leveraged on the pre-existing tools mostly derived from influenza preparedness infrastructure with minor adaptations depending on the conditions of public health response operations and international recommendations. For case management and infection prevention control pillars, COVID-19 guidelines, standard operations procedures and monitoring tools were developed separately from other epidemic prone diseases in order to meet countries requirements. Regarding logistics, guidelines for emergency procurement system to fast track procurement process without circumventing existing laws and regulations were implemented in all member states. This also facilitated the procurement of goods for the management of other outbreaks.

**Influence of the COVID-19 pandemic management on other epidemics management:** COVID-19 pandemic in West Africa has brought both positive and negative effects to the management of other epidemic prone diseases as reported by countries representatives. Positive effects included activation of crisis management committees with involvement at the highest political level, inter-ministerial and inter-agency collaborations including army health services, improved pooling of resources from various sectors, strengthening the skills of health workers at all levels in the health systems, improvement of existing monitoring system including events based surveillance/indicators based surveillance, scaling up of Surveillance Outbreak Response Management and Analysis System (SORMAS) nationwide and linking with DHIS-2 to laboratory data for easing real time reporting in Ghana and Nigeria, scaling up of molecular diagnostics among ECOWAS member states, improving equipments of health structures including laboratory systems, improving infection prevention and control systems in hospitals and communities, active Information sharing and cross border surveillance among ECOWAS member states, better awareness of non-pharmaceutical measures on management of diseases, expanded stakeholders and media network and strengthened relationship, virtual mentorships of responders in several member states and internal manufacture of consumables and goods.

The negative effects of COVID-19 pandemic to other epidemics management included overall insufficiency of personal protective equipment (and especially gowns and face masks), essential drugs, laboratory supplies and reagents, vaccines and other hospital and equipment due to closure of international borders, lack of sufficient human resources, high numbers of health worker infections which added to the lack of adequate workforce and work overload among healthcare workers, lack of community trust to government during the outbreaks response, challenges with governance and power dynamics in some countries, reduced attendance to health facilities and finally disruption of major program activities in and out of the health sector.

**Conclusion:** the COVID-19 pandemic has been a challenging experience in West Africa and has brought major changes in the public health response of infectious diseases outbreaks both in ECOWAS member states and at regional level. The experience in managing the coexistence of other epidemics with COVID-19 pandemic led the RCSDC to recommend to member states that they should reinforce regular disease surveillance for seasonal outbreaks and country specific epidemiological diseases profile while not forgetting the emerging and remerging diseases. Countries should also adapt to their local context and foster the implementation of the third generation of integrated disease surveillance and response guidelines. Countries with the support of RCSDC and other regional and international organizations should develop guidance documents on critical response pillars for improving the management the coexistence of several epidemics in one country and at the same time. In addition, health system strengthening efforts should be maintained and promoted. Finally, partners and funders should give a particular interest on supporting and funding activities intended to see these recommendations implemented.
